# Role of Proteostasis Regulation in the Turnover of Stress Granules

**DOI:** 10.3390/ijms232314565

**Published:** 2022-11-23

**Authors:** Rirong Hu, Beituo Qian, Ang Li, Yanshan Fang

**Affiliations:** 1 Interdisciplinary Research Center on Biology and Chemistry, Shanghai Institute of Organic Chemistry, Chinese Academy of Sciences, Shanghai 201203, China; 2 University of Chinese Academy of Sciences, Beijing 100049, China; 3 Guangdong-Hong Kong-Macau Institute of CNS Regeneration, Key Laboratory of CNS Regeneration of Ministry of Education, Jinan University, Guangzhou 510632, China

**Keywords:** stress granule, chaperones, UPS, autophagy, ubiquitin, VCP, G3BP

## Abstract

RNA-binding proteins (RBPs) and RNAs can form dynamic, liquid droplet-like cytoplasmic condensates, known as stress granules (SGs), in response to a variety of cellular stresses. This process is driven by liquid–liquid phase separation, mediated by multivalent interactions between RBPs and RNAs. The formation of SGs allows a temporary suspension of certain cellular activities such as translation of unnecessary proteins. Meanwhile, non-translating mRNAs may also be sequestered and stalled. Upon stress removal, SGs are disassembled to resume the suspended biological processes and restore the normal cell functions. Prolonged stress and disease-causal mutations in SG-associated RBPs can cause the formation of aberrant SGs and/or impair SG disassembly, consequently raising the risk of pathological protein aggregation. The machinery maintaining protein homeostasis (proteostasis) includes molecular chaperones and co-chaperones, the ubiquitin-proteasome system, autophagy, and other components, and participates in the regulation of SG metabolism. Recently, proteostasis has been identified as a major regulator of SG turnover. Here, we summarize new findings on the specific functions of the proteostasis machinery in regulating SG disassembly and clearance, discuss the pathological and clinical implications of SG turnover in neurodegenerative disorders, and point to the unresolved issues that warrant future exploration.

## 1. Introduction

Stress granules (SGs) are phase-separated biomolecular condensates of RNA-binding proteins (RBPs) and mRNAs, which form liquid droplet-like, membraneless cytoplasmic compartments in response to stress. The primary function of SGs is to promote cell survival in stress by providing a temporary reservoir for storing translationally stalled mRNAs, RBPs, and ribosomal proteins. The low-complexity domain contained in many SG-associated RBPs tends to be intrinsically disordered and serves as a driving force for lipid-lipid phase separation (LLPS) that initiates the assembly of SGs [1,2]. Meanwhile, the composition and concentration of RBPs, the species and abundance of RNAs, the interaction between RBPs and between RBPs and RNAs, and the post-translational modifications (PTMs) of RBPs, as well as factors in the micro-environment such as pH, ionic concentration, temperature, and metabolites can also regulate or modify the process of phase separation and SG assembly [1,3,4,5].

**Figure 1:**
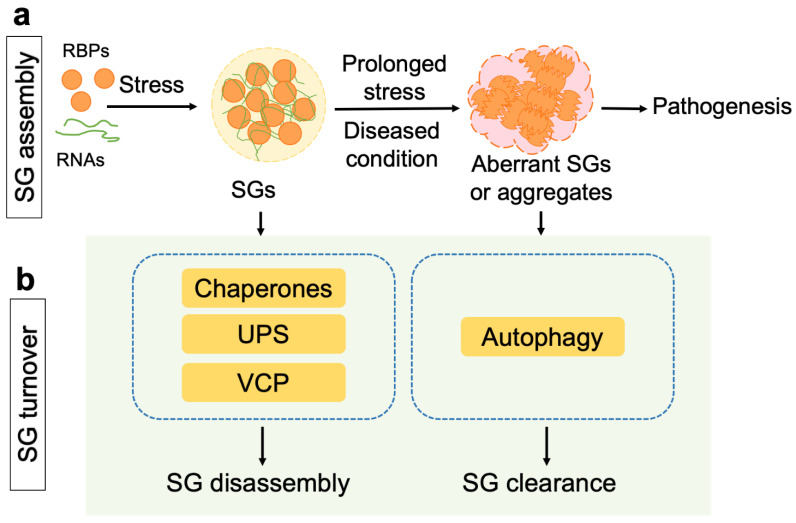
A schematic of the formation and turnover of SGs. (**a**) Cellular stress induces the assembly of SGs containing RBPs and RNAs. Under diseased conditions such as in prolonged stress or induced by pathogenic mutations, aberrant SGs and/or protein aggregation are formed in cells. (**b**) Upon removal of stress, dynamic SGs are promptly disassembled by molecular chaperones, the UPS and VCP, whereas aberrant SGs and solid protein aggregates are cleared via the autophagy pathway. Abbreviations: RBPs, RNA-binding proteins; SGs, stress granules; UPS, ubiquitin-proteasome system; VCP, valosin-containing protein.

In normal cells, SGs are promptly disassembled when stress is relieved. In diseased conditions, aberrant SG assembly and/or liquid-to-solid phase transition may occur, triggering the formation of solid protein aggregates that are considered pathogenic in neurodegenerative diseases (Figure 1a). Disease-causal mutations in the genes encoding SG-associated RBPs are shown to alter the protein properties, making them less soluble and inclined to aggregate [4,6,7]. In addition, protein misfolding is increased during cellular stress and proteostasis disturbance. Misfolded proteins appear to accumulate in SGs, making the latter lose the liquid-like dynamics and form protein aggregation [2]. Thus, while the assembly and function of SGs have been a hot topic for research in the past decade, the role of SG turnover in preventing pathological protein aggregation has become increasingly clear and the molecular mechanisms regulating SG disassembly and clearance are emerging.

Protein homeostasis (proteostasis) refers to a balanced state in which proteins are maintained in the proper conformations, concentrations, and subcellular locations so that they can execute their cellular functions to maintain the integrity and functionality of a cell [8]. A sophisticated system has evolved to regulate proteostasis in cells, which controls the entire life cycle of proteins from synthesis to disposal. The proteostasis regulation system involves a variety of components, including the translational machinery, molecular chaperones and co-chaperones, the ubiquitin-proteasome system (UPS), and the autophagy pathway. Proteostasis disturbance is evident in normal aging and is associated with age-related neurodegenerative diseases [9,10]. In particular, malfunction of the UPS and/or autophagy can lead to accumulation and aggregation of misfolded proteins and impair organelles as well as biomolecular condensates such as SGs, which may further accelerate the degeneration process [10].

The alterations of the micro-environment and chronic stress during aging can lead to SG assembly and accumulation, which in turn may promote aging and age-related diseases. The related topics have been reviewed elsewhere [4,11,12] and are not examined here, as this mini-review focuses on the recent advances in our understanding of the molecular players and mechanisms regulating SG turnover. In this review, we first go through the major players regulating the disassembly of SGs, including the molecular chaperones, the UPS, the ubiquitin-dependent segregase valosin-containing protein (VCP), and other factors. Next, we summarize the recent findings on autophagy-mediated clearance of aberrant SGs and SG-derived protein aggregation (Figure 1b). Finally, we discuss the unsolved key questions in SG turnover with the prospect of developing novel therapeutic strategies.

## 2. Proteostasis Regulation in the Disassembly of Stress Granules

### 2.1. Molecular Chaperones

Molecular chaperones are a class of proteins that assist in protein folding and re-folding as well as the assembly of protein complexes. Heat shock proteins (Hsps) are probably the most extensively studied chaperones, which are divided into sub-families according to their molecular weight, including Hsp90s, Hsp70s, Hsp40s, and small Hsps. Hsps play a vital role in refolding, degradation, and sequestration of misfolded proteins in either an ATPase-dependent or ATPase-independent manner [13]. Mutations in the genes encoding Hsps, such as *DNAJC6*, *DNAJC9,* and *HSPB1*, are reported to cause Parkinson’s disease, autosomal recessive spastic ataxia of Charlevoix-Saguenay, and CharcotMarie-Tooth neuropathy [14,15]. Furthermore, overexpression (OE) of Hsps in a variety of cell and animal models of neurodegenerative diseases is shown to reduce pathological protein aggregation [16]. In addition, recent advances in the research of chaperones have highlighted a new layer of their regulation in proteostasis and cellular homeostasis. This is related to the ability of Hsps to regulate protein phase separation and/or phase transition, thereby regulating SG disassembly and preventing misfolded proteins from accumulating in SGs (Table 1).

Molecular chaperones regulate SG disassembly and clearance. Hsp70 is one of the first Hsps shown to facilitate SG disassembly, and it plays a central role in this regulation. Upregulation of Hsp70 promotes SG disassembly and translation restoration in cells after being released from heat shock in *Drosophila melanogaster* [24], whereas deficiency of Hsp70 delays SG disassembly in both yeast [25] and mammalian cells [17,18]. Ydj1 and Sis1, two *Saccharomyces cerevisiae* Hsp40 molecular chaperones, are the co-chaperone of Hsp70, which determine the substrate specificity and enhance the ATPase activity of Hsp70. Hsp70 as well as Ydj1 and Sis1 proteins are found accumulating in SGs and the defects in the latter two reduce the disassembly and/or clearance of SGs [25]. Pharmacological activation of Hsp70 has been shown to reduce aggregation of huntingtin and alpha-synuclein. For example, YM-1 is a pharmacological mimetic of Hip (a co-chaperone that enhances binding of Hsp70 to its substrates), which could allosterically activate Hsp70 and rescue polyglutamine toxicity in a *Drosophila* model of spinobulbar muscular atrophy [26]. Likewise, activation of Hsp70 with YM-1 also modulated huntingtin proteostasis by reducing aggregation of huntingtin, which hence holds potential for treating Huntington’s disease [27]. MAL1-271, a synthetic molecule directly increasing the ATPase activity of Hsp70, reduced synuclein aggregation in a model of Parkinson’s disease [28].

As mentioned above, a fundamental role of molecular chaperones is that they facilitate protein folding and prevent the accumulation of misfolded proteins. This function is also essential for maintaining the assembly–disassembly dynamics of SGs. For instance, VER-155008, a potent small molecule inhibitor of the Hsp70 family, induces substantial SG-localized accumulation of misfolded proteins resulting in aberrant SG formation, and the disassembly of these SGs requires the functional HspB8-BAG3-Hsp70 chaperone complex [17,18]. Of note, HspB8 is a small Hsp that binds misfolded proteins and subsequently confers them to Hsp70, while BAG3 is a nucleotide exchange factor that endows the functional specificity of Hsp70. The HspB8-BAG3-Hsp70 complex not only helps with the autophagic degradation of misfolded proteins [29], but also assists in removing misfolded proteins from SGs to facilitate SG turnover [17]. Besides, another small Hsp, HspB1, has been shown to inhibit the LLPS of fused in sarcoma (FUS), which prevents the localization and association of FUS with SGs, suggesting that the LLPS capability of RBPs may be required for their partitioning in SGs [22].

In addition to regulating the LLPS of the SG-associated RBPs, a few recent studies have demonstrated that some molecular chaperones can phase separate on their own and/or co-phase separate with RBPs, thereby preventing liquid-to-solid phase transition of SGs. For example, human Hsp40 proteins such as Hdj1 (DNAJB1) and Hdj2 (DNAJA1) display an intrinsic property of LLPS, and mutations in Hdj1 that disrupt its LLPS capability decrease its co-LLPS with FUS, reduce its association with SGs, and promote maturation of FUS into solid fibrils [21]. Likewise, Hsp70 exhibits the capability to phase separate with TDP-43 [19] and with FUS [20], thereby stabilizing them in the phase-separated, liquid-like state and preventing the proceeding to toxic aggregation.

The chaperone Hsp90 is thought to function downstream of Hsp70 in regulating protein folding [30]. Although inhibition of the ATPase activity of Hsp90 barely elicits any accumulation of misfolded proteins inside SGs, Hsp90 can promote SG disassembly due to its interaction and stabilization of the dual-specificity tyrosine-phosphorylation-regulated kinase 3 (DYRK3) [23], as the active DYRK3 promotes SG disassembly and restores mTORC1 signaling and translation [23,31].

### 2.2. The Ubiquitin-Proteasome System (UPS)

The UPS is the primary ubiquitin-mediated proteolytic pathway that is responsible for the elimination of over 80% of damaged or misfolded proteins in eukaryotic cells [32]. It comprises proteasomes, ubiquitin, various protein adaptors, and enzymes that regulate ubiquitination and deubiquitination of substrate proteins. The UPS can recognize ubiquitinated misfolded proteins and subject them to proteasomes for timely degradation [33]. When the functional capacity of the UPS is impaired in diseased conditions or when misfolded proteins somehow escape from the protein quality control system, they accumulate and form pathological aggregation. Recent research in proteasome biology has demonstrated that genetic or pharmacological enhancement of the proteasome function can alleviate the neurodegenerative phenotypes in animal models [33].

The UPS also plays a pivotal role in regulating the assembly–disassembly of SGs. First, interruption of the UPS function, such as by the proteasome inhibitor MG132, induces proteostasis stress, which can elicit SG formation in cells [17,34]. Secondly, pharmacological inhibition of the ubiquitin-activating enzyme or proteasomes delays the disassembly of heat shock- and arsenite-induced SGs after the stress is relieved [35,36,37]. Thirdly, the deubiquitinases USP5 and USP13 are recruited to heat shock-induced SGs [38], and the recovery of heat shock-induced SGs is repressed with genetic depletion or pharmacological inhibition of the deubiquitinases [37,38].

The proteasome can provide an on-site degradation machinery for SG-localized misfolded proteins. For example, AN1-type zinc finger protein 1 (ZFAND1) delivers substrates to proteasomes under cellular stress [39], and it can be mobilized to arsenite-induced SGs, and it then recruits proteasomes to SGs [35]. Consistent with this function, the impairment of ZFAND1 or inhibition of proteasomes leads to the accumulation of misfolded proteins in SGs, subsequently eliciting the formation of aberrant SGs and/or protein aggregates that are subject to autophagic clearance [35]. Moreover, the proteasome foci can exhibit properties of liquid droplets [40] and stress can trigger the routing of protein clients to the degradation condensates [41].

Notably, the UPS also regulates the recruitment of RBPs into SGs. This regulation involves the PTM of SUMOylation, which covalently attaches a small ubiquitin-like modifier (SUMO) protein to the substrate proteins [42]. Protein SUMOlytion is found in SGs, and SUMOlytion of RBPs modulates the processes of both assembly and disassembly of SGs [43]. In particular, RING-type ubiquitin ligase 4 (RNF4), a mammalian SUMO-target ubiquitin ligase, mediates SUMO-primed ubiquitination and degradation of SG-associated RBPs in the nucleus during proteotoxic stress, and its impairment not only precludes the entry of a disease-associated FUS mutant into SGs but also dramatically delays SG disassembly upon stress relief [44].

In summary, the functional UPS maintains cellular proteostasis. Upon stress, cytoplasmic proteasomes are mobilized to SGs to enable on-site degradation of SG-localized proteins. Meanwhile, nuclear proteasomes are also present with misfolded RBPs in the nucleus, whose timely degradation prevents their translocation and deposition into cytoplasmic SGs.

### 2.3. Valosin-Containing Protein (VCP)/p97

The protein unfoldase VCP/p97 is an AAA+-type ATPase, which extracts ubiquitinated substrates from protein complexes, membranes, and aggregates, and subjects them to refolding or proteasomal/autophagic degradation [45]. Moreover, VCP can enhance proteasomal activity and regulate autophagosome formation and maturation. Mutations in VCP predispose humans to amyotrophic lateral sclerosis (ALS) and frontotemporal degeneration dementia (FTD), and VCP-associated ALS/FTD is characterized by ubiquitin-positive cytoplasmic inclusions containing TDP-43 [45,46].

The initial link of VCP to SG turnover came from the observation that depletion or pathogenic mutations in VCP as well as inhibition of autophagy reduced SG clearance [47]. Later, VCP was shown to be co-recruited with the 26S proteasome to SGs, which promoted SG disassembly [35]. Phosphorylation of VCP by unc-51-like autophagy activating kinases 1 and 2 (ULK1/2) activated VCP and enhanced its ability to disassemble heat shock-induced SGs; however, loss-of-function of autophagy-related genes (Atgs) such as *Atg7* did not impair SG disassembly in mouse embryonic fibroblast cells or cause the same muscle pathology elicited by *ULK1/2* deficiency in mice [48], thereby suggesting an autophagy-independent mechanism.

**Figure 2:**
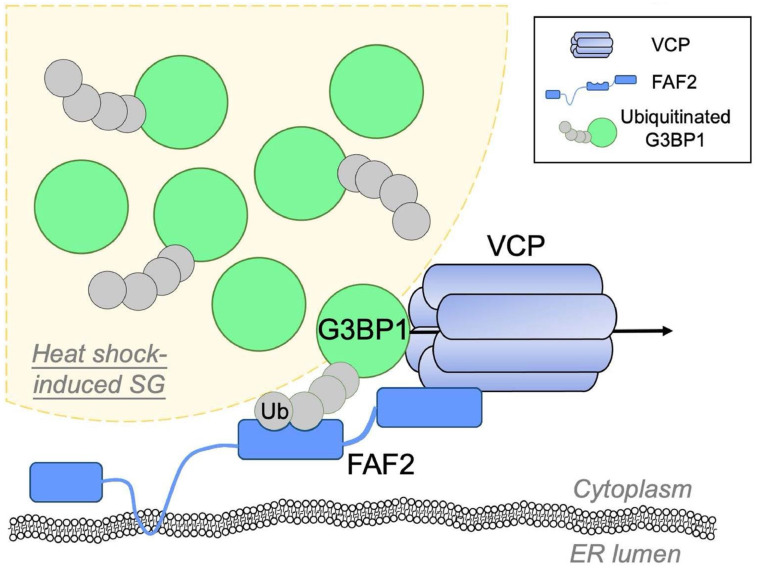
VCP extracts G3BP1 from SGs and triggers SG disassembly. G3BP is an essential protein and the core of the interaction network of SGs. Upon heat shock, G3BP1 in SGs undergoes massive ubiquitination. The ER-associated protein FAF2 recognizes ubiquitinated G3BP1 and delivers it to VCP. The “extraction” of G3BP1 from SGs by VCP triggers the dissociation of the other SG proteins, leading to the disassembly of SGs. Abbreviations: ER, endoplasmic reticulum; FAF2, FAS-associated factor 2; G3BP1, Ras GTPase-activating protein-binding protein 1; SGs, stress granules; Ub, ubiquitin; VCP, valosin-containing protein.

A recent study on the Ras-GTPase-activating protein-binding protein 1 (G3BP1) has provided novel insights into the function and molecular mechanism of VCP in regulating SG turnover [36]. Specifically, heat shock induces ubiquitination of G3BP1, and ubiquitinated G3BP1 interacts with VCP that dissociates G3BP1 from SGs. This process is mediated by the endoplasmic reticulum (ER)-associated VCP adaptor protein, FAS-associated factor 2 (FAF2), which recognizes ubiquitinated G3BP1 and recruits VCP to SGs. As a nucleating protein of the SG network of RBPs and RNAs, extraction of G3BP1 from SGs by VCP results in SG collapse and disassembly (Figure 2). In addition, SG turnover is context-dependent: acute heat shock-induced SGs are dismissed via the above-described mechanism by VCP, whereas SGs formed during prolonged heat stress are cleared via the autophagy pathway [36].

## 3. Clearance of Stress Granules and Aggregates via the Autophagy Pathway

### 3.1. Autophagy

Autophagy is a fundamental and evolutionarily conserved cellular degradation pathway, by which protein aggregates, damaged organelles, and other unnecessary or dysfunctional cellular components are removed via lysosome-mediated degradation [49]. Autophagy receptors such as p62, also known as sequestosome 1 (SQSTM1), contain LC3-interacting regions, which recognize the substrates via ubiquitin and lipid-based signals. The phagophore grows and engulfs the targets, forming a closed, double-membrane vesicle known as autophagosome that fuses with a lysosome for degradation and recycling [50]. Autophagy is crucial for stress adaptation and proteostasis regulation [51], and autophagyic and endolysosomal dysfunction is linked to various human diseases [52]. Furthermore, multiple lines of genetic and pharmacological evidence have demonstrated the prominent role of autophagy in SG clearance [47,53,54,55,56,57]. Thus, pharmacological activation of autophagy has been proposed as a potential therapeutic means to restore proteostasis and exert beneficial effects in neurodegenerative disorders [58].

### 3.2. p62/Sequestosome 1 (SQSTM1)

Delivery of SGs to autophagic degradation relies on the autophagy receptors, such as p62/SQSTM1. Notably, *SQSTM1* is a causative gene in patients with ALS [59] and FTD [60,61], and its protein p62 is found in the pathological protein inclusion in patients with ALS/FTD [62,63,64] and in SGs colocalized with the autophagosome marker LC3-II [35]. The association of p62 with SGs is enhanced in persisting SGs [65] and SGs containing an ALS/FTD-linked FUS mutant [53]. Meanwhile, it is shown that a K63 polyubiquitin (poly-Ub) chain can induce p62 phase separation in vivo and in vitro, which recruits LC3-II and fosters autophagic degradation of p62 [66]. Given that chaperones such as Hsp27, Hsp40, and Hsp70 are recruited to SGs by co-phase separation with RBPs [19,20,21,22], it is possible that p62 is partitioned into SGs by poly-Ub-induced phase separation that promotes autophagic clearance of p62-associated aberrant SGs.

p62 can recognize methylated proteins in addition to ubiquitinated proteins. SG-associated RBPs such as FUS are symmetrically methylated on arginines, which are recognized by another ALS-linked protein survival motor neuron (SMN). SMN then brings p62 to arginine-methylated RBPs, triggering the p62-mediated autophagic clearance of SGs [56]. Patients with *C9ORF72* repeat expansions, the major genetic cause of ALS [67], accumulate arginine-dimethylated proteins that co-localize with p62, whereas mice lacking p62 accumulate arginine-methylated proteins [56]. These findings suggest that C9ORF72 associates with the autophagy receptor p62 and affects autophagy-dependent elimination of SGs. Given that SGs are rich in arginine-containing RBPs [68,69], protein methylation at arginines may serve as a unique signal for p62-mediated autophagic clearance of SGs.

### 3.3. Chaperonin-Containing TCP-1 Subunit 2 (CCT2)

The TRiC (chaperonin TCP-1 ring complex) subunit chaperonin-containing TCP-1 subunit 2 (CCT2) is a newly identified aggrephagy receptor in mammals, which specifically mediates the elimination of solid aggregates, but not liquid-like condensates, via the autophagy pathway. Additionally, it is shown that CCT2 functions independently of ubiquitin or the TRiC complex to facilitate the autophagic clearance of solid protein aggregates [70].

Although a direct role of CCT2 in SG clearance has yet to be demonstrated, multiple lines of evidence suggest its possible involvement. First, the subunits of TRiC are abundantly expressed in SGs [69]. Secondly, the TRiC complex functions to prevent protein aggregation [71] and CCT2 is associated with aggregation-prone proteins [72,73], while SGs are enriched with aggregation-prone RBPs [74,75,76,77,78,79]. Thirdly, CCT2 interacts with FUS and OE of CCT2 enhances autophagic clearance of mutant FUS only when it becomes solid aggregates [70]. Thus, it is possible that different autophagy receptors govern the clearance of SGs at different phases. p62 recognizes protein ubiquitination and arginine methylation in aberrant, less dynamic SGs, whereas CCT2 mediates the clearance of solid protein aggregates that are derived from liquid-to-solid phase transition of SGs.

## 4. Concluding Remarks

**Figure 3:**
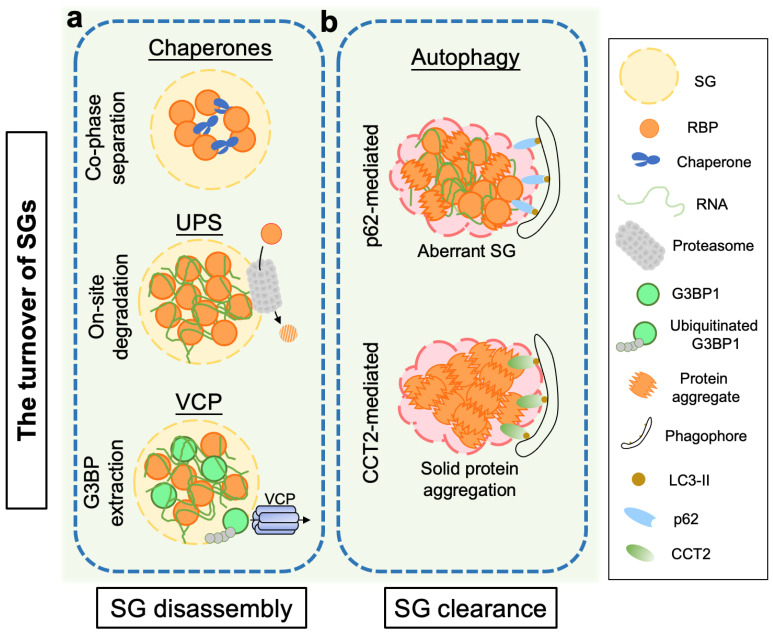
The turnover of SGs—disassembly and clearance. (**a**) The disassembly of liquid-like, dynamic SGs are mediated by molecular chaperones, the UPS and VCP. Chaperones prevent liquid-to-solid phase transition of SGs by co-phase separation with SG-associated RBPs; the proteasome provides an on-site degradation machinery for elimination of misfolded proteins in SGs; and VCP dissociates ubiquitinated G3BP1 from SGs, trigging SG disassembly. (**b**) Aberrant, persisting SGs and solid protein aggregates are recognized by the autophagy receptors p62 and CCT2, respectively, which are subsequently eliminated by autophagic degradation. Abbreviations: CCT2, chaperonin-containing TCP-1 subunit 2; G3BP1, Ras GTPase-activating protein-binding protein 1; RBP, RNA-binding protein; SGs, stress granules; UPS, ubiquitin-proteasome system; VCP, valosin-containing protein.

As summarized in Figure 3, to maintain the liquid-like, dynamic property of the phase separated SGs, various factors and pathways in cells participate in the regulation of the SG disassembly, such as the molecular chaperones (which co-phase separate with SG-associated RBPs and prevent the liquid-to-solid phase transition of SGs), the UPS (which performs on-site degradation of SG-localized misfolded proteins), and VCP (which extracts G3BP1 from SGs and causes the subsequent SG disassembly). When aberrant SGs form or SGs become solid aggregation, the autophagy receptors p62 and CCT2 recognize and target aberrant SGs and solid aggregates for autophagy-mediated clearance, respectively.

Although the UPS enables a highly efficient degradation of SG-localized misfolded proteins, it is unclear how exactly these proteins are recognized within SGs and sorted out for degradation by the UPS. G3BP1/2 are so far the only SG-associated RBPs that have been proven essential for SG assembly, as their knockout disables the formation of SGs [80,81,82] and their removal by VCP is sufficient to trigger SG disassembly [36]. What makes G3BP1/2 so unique, sequence- and structure-wise? Does VCP extract any other SG-associated protein or any protein associated with other ribonucleoprotein granules such as P body and nuclear bodies? The autophagy pathway plays a major role in the clearance of aberrant SGs and SG-derived protein aggregation. In chronic neurodegenerative diseases, however, the liquid-to-phase transition and maturation of SGs to pathological aggregation are often gradually developed. So, how do cells surveil the states of SGs and promptly recognize the aberrant SGs and aggregation? Along the line, protein ubiquitination appears to be a molecular signal used for both degradation of misfolded proteins and turnover of aberrant SGs. As such, when an SG-associated RBP, e.g., G3BP1/2, is ubiquitinated, how do cells distinguish whether the ubiquitination of G3BP1/2 is to label it for routine protein degradation or to signal the SGs for disassembly and/or clearance?

Finally, given the association of the proteostasis regulation and SGs in neurodegenerative diseases, there have been therapeutic attempts targeting molecular chaperones, the UPS, and autophagy [16,33,58]. With recent advances in the understanding of the regulation of SG disassembly and clearance, novel intervention strategies should be considered, such as to enhance co-LLPS of Hsps with RBPs, to improve the function of VCP in removing G3BP1/2, and to promote precise recognition and efficient clearance of aberrant SGs via autophagy. Together, we expect that further elucidation of the regulatory mechanisms of SG turnover will assist in the development of effective treatments for neurodegenerative diseases.

## Figures and Tables

**Table 1:** Chaperones in SG disassembly.

Name	Function in SG Disassembly	References
Hsp70	Prevent misfolded proteins from accumulation	[17,18]
	Prevent liquid-to-solid phase transition of FUS and TDP-43	[19,20]
Hdj1	Prevent liquid-to-solid phase transition of FUS	[21]
HspB8	Prevent misfolded proteins from accumulation	[17]
HspB1	Inhibit LLPS of FUS and its association with SGs	[22]
Hsp90	Enhance DYRK3 activity that promotes SG disassembly	[23]

## References

[1] Glauninger H., Wong Hickernell C.J., Bard J.A.M., Drummond D.A. (2022). Stressful steps: Progress and challenges in understanding stress-induced mRNA condensation and accumulation in stress granules. Mol. Cell.

[2] Alberti S., Hyman A.A. (2021). Biomolecular condensates at the nexus of cellular stress, protein aggregation disease and ageing. Nat. Rev. Mol. Cell Biol..

[3] Hofweber M., Dormann D. (2019). Friend or foe-Post-translational modifications as regulators of phase separation and RNP granule dynamics. J. Biol. Chem..

[4] Wolozin B., Ivanov P. (2019). Stress granules and neurodegeneration. Nat. Rev. Neurosci..

[5] Krause L.J., Herrera M.G., Winklhofer K.F. (2022). The Role of Ubiquitin in Regulating Stress Granule Dynamics. Front. Physiol..

[6] Ivanov P., Kedersha N., Anderson P. (2019). Stress Granules and Processing Bodies in Translational Control. Cold Spring Harb. Perspect. Biol..

[7] Zhang Y., Gu J., Sun Q. (2021). Aberrant Stress Granule Dynamics and Aggrephagy in ALS Pathogenesis. Cells.

[8] Clausen L., Abildgaard A.B., Gersing S.K., Stein A., Lindorff-Larsen K., Hartmann-Petersen R. (2019). Protein stability and degradation in health and disease. Adv. Protein Chem. Struct. Biol..

[9] Hou Y., Dan X., Babbar M., Wei Y., Hasselbalch S.G., Croteau D.L., Bohr V.A. (2019). Ageing as a risk factor for neurodegenerative disease. Nat. Rev. Neurol..

[10] Hipp M.S., Kasturi P., Hartl F.U. (2019). The proteostasis network and its decline in ageing. Nat. Rev. Mol. Cell Biol..

[11] Cao X., Jin X., Liu B. (2020). The involvement of stress granules in aging and aging-associated diseases. Aging Cell.

[12] Cadena Sandoval M., Heberle A.M., Rehbein U., Barile C., Ramos Pittol J.M., Thedieck K. (2021). mTORC1 Crosstalk with Stress Granules in Aging and Age-Related Diseases. Front. Aging.

[13] Rosenzweig R., Nillegoda N.B., Mayer M.P., Bukau B. (2019). The Hsp70 chaperone network. Nat. Rev. Mol. Cell Biol..

[14] Zarouchlioti C., Parfitt D.A., Li W., Gittings L.M., Cheetham M.E. (2018). DNAJ Proteins in neurodegeneration: Essential and protective factors. Philos. Trans. R. Soc. Lond. B Biol. Sci..

[15] Vendredy L., Adriaenssens E., Timmerman V. (2020). Small heat shock proteins in neurodegenerative diseases. Cell Stress Chaperones.

[16] Kampinga H.H., Bergink S. (2016). Heat shock proteins as potential targets for protective strategies in neurodegeneration. Lancet Neurol..

[17] Ganassi M., Mateju D., Bigi I., Mediani L., Poser I., Lee H.O., Seguin S.J., Morelli F.F., Vinet J., Leo G. (2016). A Surveillance Function of the HSPB8-BAG3-HSP70 Chaperone Complex Ensures Stress Granule Integrity and Dynamism. Mol. Cell.

[18] Mateju D., Franzmann T.M., Patel A., Kopach A., Boczek E.E., Maharana S., Lee H.O., Carra S., Hyman A.A., Alberti S. (2017). An aberrant phase transition of stress granules triggered by misfolded protein and prevented by chaperone function. EMBO J..

[19] Gu J., Wang C., Hu R., Li Y., Zhang S., Sun Y., Wang Q., Li D., Fang Y., Liu C. (2021). Hsp70 chaperones TDP-43 in dynamic, liquid-like phase and prevents it from amyloid aggregation. Cell Res..

[20] Li Y., Gu J., Wang C., Hu J., Zhang S., Liu C., Zhang S., Fang Y., Li D. (2022). Hsp70 exhibits a liquid-liquid phase separation ability and chaperones condensed FUS against amyloid aggregation. iScience.

[21] Gu J., Liu Z., Zhang S., Li Y., Xia W., Wang C., Xiang H., Liu Z., Tan L., Fang Y. (2020). Hsp40 proteins phase separate to chaperone the assembly and maintenance of membraneless organelles. Proc. Natl. Acad. Sci. USA.

[22] Liu Z., Zhang S., Gu J., Tong Y., Li Y., Gui X., Long H., Wang C., Zhao C., Lu J. (2020). Hsp27 chaperones FUS phase separation under the modulation of stress-induced phosphorylation. Nat. Struct. Mol. Biol..

[23] Mediani L., Antoniani F., Galli V., Vinet J., Carra A.D., Bigi I., Tripathy V., Tiago T., Cimino M., Leo G. (2021). Hsp90-mediated regulation of DYRK3 couples stress granule disassembly and growth via mTORC1 signaling. EMBO Rep..

[24] Cherkasov V., Hofmann S., Druffel-Augustin S., Mogk A., Tyedmers J., Stoecklin G., Bukau B. (2013). Coordination of translational control and protein homeostasis during severe heat stress. Curr. Biol..

[25] Walters R.W., Muhlrad D., Garcia J., Parker R. (2015). Differential effects of Ydj1 and Sis1 on Hsp70-mediated clearance of stress granules in Saccharomyces cerevisiae. RNA.

[26] Wang A.M., Miyata Y., Klinedinst S., Peng H.M., Chua J.P., Komiyama T., Li X., Morishima Y., Merry D.E., Pratt W.B. (2013). Activation of Hsp70 reduces neurotoxicity by promoting polyglutamine protein degradation. Nat. Chem. Biol..

[27] Pinho B.R., Almeida L.M., Duchen M.R., Oliveira J.M.A. (2021). Allosteric activation of Hsp70 reduces mutant huntingtin levels, the clustering of N-terminal fragments, and their nuclear accumulation. Life Sci..

[28] Chiang A.N., Liang M., Dominguez-Meijide A., Masaracchia C., Goeckeler-Fried J.L., Mazzone C.S., Newhouse D.W., Kendsersky N.M., Yates M.E., Manos-Turvey A. (2019). Synthesis and evaluation of esterified Hsp70 agonists in cellular models of protein aggregation and folding. Bioorg. Med. Chem..

[29] Carra S., Seguin S.J., Lambert H., Landry J. (2008). HspB8 chaperone activity toward poly(Q)-containing proteins depends on its association with Bag3, a stimulator of macroautophagy. J. Biol. Chem..

[30] Moran Luengo T., Mayer M.P., Rudiger S.G.D. (2019). The Hsp70-Hsp90 Chaperone Cascade in Protein Folding. Trends Cell Biol..

[31] Wippich F., Bodenmiller B., Trajkovska M.G., Wanka S., Aebersold R., Pelkmans L. (2013). Dual specificity kinase DYRK3 couples stress granule condensation/dissolution to mTORC1 signaling. Cell.

[32] Collins G.A., Goldberg A.L. (2017). The Logic of the 26S Proteasome. Cell.

[33] Wang X., Wang H. (2020). Priming the Proteasome to Protect against Proteotoxicity. Trends Mol. Med..

[34] Mazroui R., Di Marco S., Kaufman R.J., Gallouzi I.E. (2007). Inhibition of the ubiquitin-proteasome system induces stress granule formation. Mol. Biol. Cell..

[35] Turakhiya A., Meyer S.R., Marincola G., Bohm S., Vanselow J.T., Schlosser A., Hofmann K., Buchberger A. (2018). ZFAND1 Recruits p97 and the 26S Proteasome to Promote the Clearance of Arsenite-Induced Stress Granules. Mol. Cell.

[36] Gwon Y., Maxwell B.A., Kolaitis R.-M., Zhang P., Kim H.J., Taylor J.P. (2021). Ubiquitination of G3BP1 mediates stress granule disassembly in a context-specific manner. Science.

[37] Tolay N., Buchberger A. (2021). Comparative profiling of stress granule clearance reveals differential contributions of the ubiquitin system. Life Sci. Alliance.

[38] Xie X., Matsumoto S., Endo A., Fukushima T., Kawahara H., Saeki Y., Komada M. (2018). Deubiquitylases USP5 and USP13 are recruited to and regulate heat-induced stress granules through their deubiquitylating activities. J. Cell Sci..

[39] Sa-Moura B., Funakoshi M., Tomko R.J., Dohmen R.J., Wu Z., Peng J., Hochstrasser M. (2013). A conserved protein with AN1 zinc finger and ubiquitin-like domains modulates Cdc48 (p97) function in the ubiquitin-proteasome pathway. J. Biol. Chem..

[40] Yasuda S., Tsuchiya H., Kaiho A., Guo Q., Ikeuchi K., Endo A., Arai N., Ohtake F., Murata S., Inada T. (2020). Stress- and ubiquitylation-dependent phase separation of the proteasome. Nature.

[41] Carrettiero D.C., Almeida M.C., Longhini A.P., Rauch J.N., Han D., Zhang X., Najafi S., Gestwicki J.E., Kosik K.S. (2022). Stress routes clients to the proteasome via a BAG2 ubiquitin-independent degradation condensate. Nat. Commun..

[42] Vertegaal A.C.O. (2022). Signalling mechanisms and cellular functions of SUMO. Nat. Rev. Mol. Cell Biol..

[43] Marmor-Kollet H., Siany A., Kedersha N., Knafo N., Rivkin N., Danino Y.M., Moens T.G., Olender T., Sheban D., Cohen N. (2020). Spatiotemporal Proteomic Analysis of Stress Granule Disassembly Using APEX Reveals Regulation by SUMOylation and Links to ALS Pathogenesis. Mol. Cell.

[44] Keiten-Schmitz J., Wagner K., Piller T., Kaulich M., Alberti S., Muller S. (2020). The Nuclear SUMO-Targeted Ubiquitin Quality Control Network Regulates the Dynamics of Cytoplasmic Stress Granules. Mol. Cell.

[45] van den Boom J., Meyer H. (2018). VCP/p97-Mediated Unfolding as a Principle in Protein Homeostasis and Signaling. Mol. Cell.

[46] Ferrari V., Cristofani R., Tedesco B., Crippa V., Chierichetti M., Casarotto E., Cozzi M., Mina F., Piccolella M., Galbiati M. (2022). Valosin Containing Protein (VCP): A Multistep Regulator of Autophagy. Int. J. Mol. Sci..

[47] Buchan J.R., Kolaitis R.M., Taylor J.P., Parker R. (2013). Eukaryotic stress granules are cleared by autophagy and Cdc48/VCP function. Cell.

[48] Wang B., Maxwell B.A., Joo J.H., Gwon Y., Messing J., Mishra A., Shaw T.I., Ward A.L., Quan H., Sakurada S.M. (2019). ULK1 and ULK2 Regulate Stress Granule Disassembly Through Phosphorylation and Activation of VCP/p97. Mol. Cell.

[49] Levine B., Kroemer G. (2019). Biological Functions of Autophagy Genes: A Disease Perspective. Cell.

[50] Pohl C., Dikic I. (2019). Cellular quality control by the ubiquitin-proteasome system and autophagy. Science.

[51] Klionsky D.J., Petroni G., Amaravadi R.K., Baehrecke E.H., Ballabio A., Boya P., Bravo-San Pedro J.M., Cadwell K., Cecconi F., Choi A.M.K. (2021). Autophagy in major human diseases. EMBO J..

[52] Menzies F.M., Fleming A., Rubinsztein D.C. (2015). Compromised autophagy and neurodegenerative diseases. Nat. Rev. Neurosci..

[53] Ryu H.H., Jun M.H., Min K.J., Jang D.J., Lee Y.S., Kim H.K., Lee J.A. (2014). Autophagy regulates amyotrophic lateral sclerosis-linked fused in sarcoma-positive stress granules in neurons. Neurobiol. Aging.

[54] Krisenko M.O., Higgins R.L., Ghosh S., Zhou Q., Trybula J.S., Wang W.H., Geahlen R.L. (2015). Syk Is Recruited to Stress Granules and Promotes Their Clearance through Autophagy. J. Biol. Chem..

[55] Rodriguez-Ortiz C.J., Flores J.C., Valenzuela J.A., Rodriguez G.J., Zumkehr J., Tran D.N., Kimonis V.E., Kitazawa M. (2016). The Myoblast C2C12 Transfected with Mutant Valosin-Containing Protein Exhibits Delayed Stress Granule Resolution on Oxidative Stress. Am. J. Pathol..

[56] Chitiprolu M., Jagow C., Tremblay V., Bondy-Chorney E., Paris G., Savard A., Palidwor G., Barry F.A., Zinman L., Keith J. (2018). A complex of C9ORF72 and p62 uses arginine methylation to eliminate stress granules by autophagy. Nat. Commun..

[57] Anderson E.N., Gochenaur L., Singh A., Grant R., Patel K., Watkins S., Wu J.Y., Pandey U.B. (2018). Traumatic injury induces stress granule formation and enhances motor dysfunctions in ALS/FTD models. Hum. Mol. Genet..

[58] Scrivo A., Bourdenx M., Pampliega O., Cuervo A.M. (2018). Selective autophagy as a potential therapeutic target for neurodegenerative disorders. Lancet Neurol..

[59] Yang Y., Tang L., Zhang N., Pan L., Hadano S., Fan D. (2015). Six SQSTM1 mutations in a Chinese amyotrophic lateral sclerosis cohort. Amyotroph. Lateral Scler. Front. Degener..

[60] Le Ber I., Camuzat A., Guerreiro R., Bouya-Ahmed K., Bras J., Nicolas G., Gabelle A., Didic M., De Septenville A., Millecamps S. (2013). SQSTM1 mutations in French patients with frontotemporal dementia or frontotemporal dementia with amyotrophic lateral sclerosis. JAMA Neurol..

[61] Van der Zee J., Van Langenhove T., Kovacs G.G., Dillen L., Deschamps W., Engelborghs S., Matej R., Vandenbulcke M., Sieben A., Dermaut B. (2014). Rare mutations in SQSTM1 modify susceptibility to frontotemporal lobar degeneration. Acta Neuropathol..

[62] Arai T., Nonaka T., Hasegawa M., Akiyama H., Yoshida M., Hashizume Y., Tsuchiya K., Oda T., Ikeda K. (2003). Neuronal and glial inclusions in frontotemporal dementia with or without motor neuron disease are immunopositive for p62. Neurosci. Lett..

[63] Hiji M., Takahashi T., Fukuba H., Yamashita H., Kohriyama T., Matsumoto M. (2008). White matter lesions in the brain with frontotemporal lobar degeneration with motor neuron disease: TDP-43-immunopositive inclusions co-localize with p62, but not ubiquitin. Acta Neuropathol..

[64] Mizuno Y., Amari M., Takatama M., Aizawa H., Mihara B., Okamoto K. (2006). Immunoreactivities of p62, an ubiqutin-binding protein, in the spinal anterior horn cells of patients with amyotrophic lateral sclerosis. J. Neurol. Sci..

[65] Zhang P., Fan B., Yang P., Temirov J., Messing J., Kim H.J., Taylor J.P. (2019). Chronic optogenetic induction of stress granules is cytotoxic and reveals the evolution of ALS-FTD pathology. eLife.

[66] Sun D., Wu R., Zheng J., Li P., Yu L. (2018). Polyubiquitin chain-induced p62 phase separation drives autophagic cargo segregation. Cell Res..

[67] Balendra R., Isaacs A.M. (2018). C9orf72-mediated ALS and FTD: Multiple pathways to disease. Nat. Rev. Neurol..

[68] Bedford M.T., Clarke S.G. (2009). Protein arginine methylation in mammals: Who, what, and why. Mol. Cell.

[69] Jain S., Wheeler J.R., Walters R.W., Agrawal A., Barsic A., Parker R. (2016). ATPase-Modulated Stress Granules Contain a Diverse Proteome and Substructure. Cell.

[70] Ma X., Lu C., Chen Y., Li S., Ma N., Tao X., Li Y., Wang J., Zhou M., Yan Y.B. (2022). CCT2 is an aggrephagy receptor for clearance of solid protein aggregates. Cell.

[71] Grantham J. (2020). The Molecular Chaperone CCT/TRiC: An Essential Component of Proteostasis and a Potential Modulator of Protein Aggregation. Front. Genet..

[72] David D.C., Ollikainen N., Trinidad J.C., Cary M.P., Burlingame A.L., Kenyon C. (2010). Widespread protein aggregation as an inherent part of aging in C. elegans. PLoS Biol..

[73] Kim Y.E., Hosp F., Frottin F., Ge H., Mann M., Hayer-Hartl M., Hartl F.U. (2016). Soluble Oligomers of PolyQ-Expanded Huntingtin Target a Multiplicity of Key Cellular Factors. Mol. Cell.

[74] Lin Y., Protter D.S., Rosen M.K., Parker R. (2015). Formation and Maturation of Phase-Separated Liquid Droplets by RNA-Binding Proteins. Mol. Cell.

[75] Molliex A., Temirov J., Lee J., Coughlin M., Kanagaraj A.P., Kim H.J., Mittag T., Taylor J.P. (2015). Phase separation by low complexity domains promotes stress granule assembly and drives pathological fibrillization. Cell.

[76] Murakami T., Qamar S., Lin J.Q., Schierle G.S., Rees E., Miyashita A., Costa A.R., Dodd R.B., Chan F.T., Michel C.H. (2015). ALS/FTD Mutation-Induced Phase Transition of FUS Liquid Droplets and Reversible Hydrogels into Irreversible Hydrogels Impairs RNP Granule Function. Neuron.

[77] Patel A., Lee H.O., Jawerth L., Maharana S., Jahnel M., Hein M.Y., Stoynov S., Mahamid J., Saha S., Franzmann T.M. (2015). A Liquid-to-Solid Phase Transition of the ALS Protein FUS Accelerated by Disease Mutation. Cell.

[78] Murray D.T., Kato M., Lin Y., Thurber K.R., Hung I., McKnight S.L., Tycko R. (2017). Structure of FUS Protein Fibrils and Its Relevance to Self-Assembly and Phase Separation of Low-Complexity Domains. Cell.

[79] Cao Q., Boyer D.R., Sawaya M.R., Ge P., Eisenberg D.S. (2019). Cryo-EM structures of four polymorphic TDP-43 amyloid cores. Nat. Struct. Mol. Biol..

[80] Guillen-Boixet J., Kopach A., Holehouse A.S., Wittmann S., Jahnel M., Schlussler R., Kim K., Trussina I., Wang J., Mateju D. (2020). RNA-Induced Conformational Switching and Clustering of G3BP Drive Stress Granule Assembly by Condensation. Cell.

[81] Yang P., Mathieu C., Kolaitis R.M., Zhang P., Messing J., Yurtsever U., Yang Z., Wu J., Li Y., Pan Q. (2020). G3BP1 Is a Tunable Switch that Triggers Phase Separation to Assemble Stress Granules. Cell.

[82] Sanders D.W., Kedersha N., Lee D.S.W., Strom A.R., Drake V., Riback J.A., Bracha D., Eeftens J.M., Iwanicki A., Wang A. (2020). Competing Protein-RNA Interaction Networks Control Multiphase Intracellular Organization. Cell.

